# Letter to the Editor: regarding “Predictive validity of a novel non-invasive estimation of effective shunt fraction in critically ill patients”

**DOI:** 10.1186/s40635-020-00318-y

**Published:** 2020-07-02

**Authors:** Philip J. Peyton, Luke R. Fletcher

**Affiliations:** 1 Department of Anaesthesia, Austin Health, 145 Studley Road, Heidelberg, VIC 3084 Australia; 2 Anaesthesia, Perioperative and Pain Medicine Unit (APPMU), Melbourne Medical School, University of Melbourne, Parkville, VIC Australia

## To the Editor,

We read with interest the recent paper on “a novel non-invasive estimation of effective shunt fraction” (Chang et al. Intensive Care Medicine Experimental (2019) 7:49) [1]. The study retrospectively investigated the validity of various oxygenation indices in predicting the change in PaO_2_ in response to a change in FIO_2_ in critically ill patients. In particular, they investigated a method to estimate shunt fraction which substitutes in the denominator of the classical shunt equation of Berggren the term for mixed venous oxygen content (CvO_2_) with a term derived from transposition of the direct oxygen Fick equation which incorporates the ratio of oxygen uptake rate to pulmonary blood flow (VO_2_/Q). Their method then assumes that VO_2_ and Q may be treated as constants.

We firstly observe that the equation they present (Eq. 2) is wrong. However, this error was not reproduced in the subsequent derivations in their Appendix 1 (Eqs. 3 and 4) and is unlikely to have invalidated their findings.

We further point out that the correct derivation of the non-invasive equation was originally presented by our group in 2004–5 in a prospective series of studies in patients undergoing cardiac surgery [2, 3], and is as follows:

Qs/Qt = (Cc’O_2_ − CaO_2_)/(Cc’O_2_ − CaO_2_ + VO_2_/Qt)

We were able to demonstrate good agreement between this non-invasive shunt fraction measurement and the classical invasive shunt equation of Berggren, and also demonstrated how a measurement of non-shunt pulmonary blood flow could be used to produce the same result. This concept is particularly attractive because of the availability nowadays of minimally invasive estimates of effective pulmonary blood flow or cardiac output from a variety of technologies, without mixed venous blood sampling or right heart catheterisation. However, a key purpose of our studies was to determine the robustness of the concept to inherent imprecision in measurement of either VO_2_ or Q under clinical conditions.

Chang et al. showed that the non-invasive shunt estimate provides better prediction of change in PaO_2_ with change in FIO_2_ than simple tension-based oxygenation indices, in situations where shunt, VO_2_ and Q are assumed to be unchanged. Under such circumstances, when comparing two steady states (A and B) with the same shunt fraction at different FIO_2_, the above equation will indeed eliminate VO_2_ and Q, and may be reduced to the following:

Cc’O_2_^A^ – CaO_2_^A^ = Cc’O_2_^B^ – CaO_2_^B^

Chang et al. have therefore demonstrated that the alveolar-arterial difference in oxygen content must remain constant, at any FIO_2_, if shunt fraction, VO_2_ and Q are unchanged. We certainly agree that this is a useful application of the non-invasive shunt principle to patient management in clinical care, but would caution that using it to estimate an absolute value for shunt fraction without measurement of VO_2_ or Q may lead to error in the calculation, and may be unsatisfactory for other purposes, such as physiological research.

Yours sincerely,

Philip Peyton

Luke Fletcher

## Data Availability

Not applicable.

## Abbreviations

CaO_2_: Arterial oxygen content (L/L)
Cc’O_2_: Pulmonary capillary oxygen content (L/L)
CvO_2_: Mixed venous oxygen content (L/L)
FIO_2_: Inspired oxygen concentration (%)
PaO_2_: Partial pressure of arterial oxygen (mmHg)
Q, Qt: Cardiac output (L/min)
Qs/Qt: Pulmonary shunt fraction (%)
VO_2_: Oxygen consumption (L/min)

## References

[1] Chang EM, Bretherick A, Drummond GB, Baillie K (2019). Predictive validity of a novel non-invasive estimation of effective shunt fraction in critically ill patients. Intensive Care Medicine Experimental.

[2] Peyton P, Robinson G, McCall P, Thompson B (2004). Non-invasive measurement of intrapulmonary shunting. J Cardiothorac Vasc Anesth.

[3] Peyton P, Poustie S, Robinson G, Penny D, Thompson B (2005). Non-invasive measurement of intrapulmonary shunt during inert gas rebreathing. Physiol Meas.

